# Active Sites of Mixed-Metal Core–Shell Oxygen Evolution Reaction Catalysts: FeO_4_ Sites on Ni Cores or NiN_4_ Sites in C Shells?

**DOI:** 10.1021/acsomega.3c09920

**Published:** 2024-06-07

**Authors:** Sung Soo Lim, Arumugam Sivanantham, Changwon Choi, Sangaraju Shanmugam, Yves Lansac, Yun Hee Jang

**Affiliations:** † Department of Energy Science and Engineering, DGIST, Daegu 42988, Korea; ‡ GREMAN, UMR 7347, Université de Tours, CNRS, INSA CVL, 37200 Tours, France; § LPS, CNRS UMR 8502, Université Paris-Saclay, 91405 Orsay, France

## Abstract

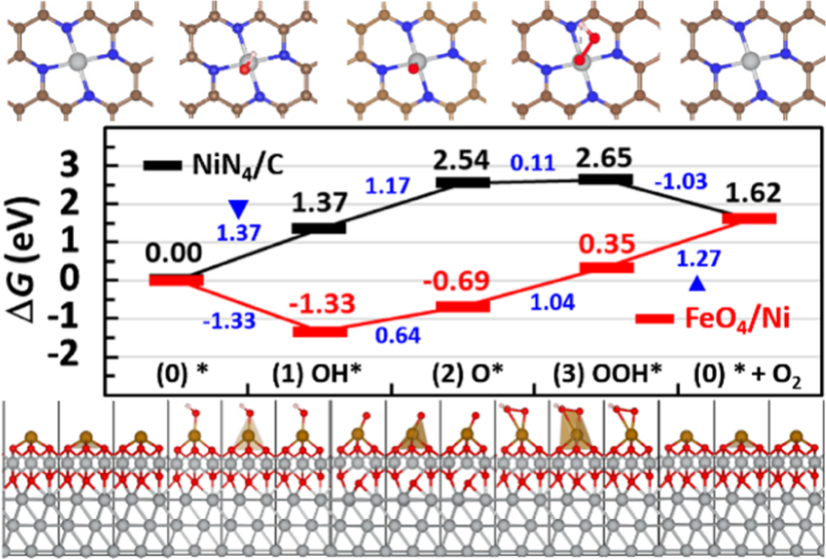

Water electrolysis for clean hydrogen production requires high-activity, high-stability, and low-cost catalysts for its particularly sluggish half-reaction, the oxygen evolution reaction (OER). Currently, the most promising of such catalysts working in alkaline conditions is a core–shell nanostructure, NiFe@NC, whose Fe-doped Ni (NiFe) nanoparticles are encapsulated and interconnected by N-doped graphitic carbon (NC) layers, but the exact OER mechanism of these catalysts is still unclear, and even the location of the OER active site, either on the core side or on the shell side, is still debated. Therefore, we herein derive a plausible active-site model for each side based on various experimental evidence and density functional theory calculations and then build OER free-energy diagrams on both sides to determine the active-site location. The core-side model is an FeO_4_-type (rather than NiO_4_-type) active site where an Fe atom sits on Ni oxide layers grown on top of the core surface during catalyst activation, whose facile dissolution provides an explanation for the activity loss of such catalysts directly exposed to the electrolyte. The shell-side model is a NiN_4_-type (rather than FeN_4_-type) active site where a Ni atom is intercalated into the porphyrin-like N_4_C site of the NC shell during catalyst synthesis. Their OER free-energy diagrams indicate that both sites require similar amounts of overpotentials, despite a complete shift in their potential-determining steps, i.e., the final O_2_ evolution from the oxophilic Fe on the core and the initial OH adsorption to the hydrophobic shell. We conclude that the major active sites are located on the core, but the NC shell not only protects the vulnerable FeO_4_ active sites on the core from the electrolyte but also provides independent active sites, owing to the N doping.

## 1 Introduction

Hydrogen can be a clean energy source slowing down climate change^1,2^ if current steam-reforming production is replaced by carbon-free electrochemical water splitting (2H_2_O → 2H_2_ + O_2_, Δ*G*_0_ = 4.92 eV).^1^ However, this reaction consists of two unfavorable endothermic half-cell reactions,^3^ i.e., a sluggish two-electron reduction process of hydrogen evolution reaction (HER; 2H_2_O + 2e^–^ → H_2_ + 2OH^–^; *E*_0_ = −0.83 V at pH 14) at the cathode^4^ and an even more sluggish four-electron oxidation process of oxygen evolution reaction (OER; 4OH^–^ → O_2_ + 2H_2_O + 4e^–^; *E*_0_ = 0.40 V at pH 14) at the anode^5−8^
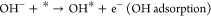
1
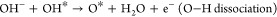
2
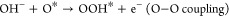
3
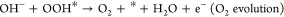
4where * represents the bare adsorption sites on catalytic electrode surfaces and *E*_0_ denotes the standard reduction potential. Relatively low OER overpotentials and Tafel slopes have been reported on RuO_2_ and IrO_2_ catalysts,^6^ but the high cost of such noble metal catalysts impedes their commercialization. Thus, there has been an extensive search for non-noble-metal OER catalysts with low cost, high activity, and high stability.^6,9−11^

**Figure 1:**
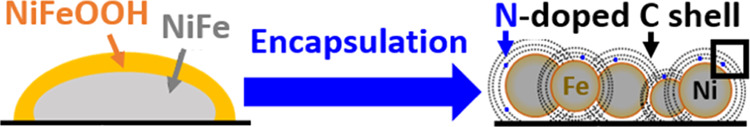
Ni(Fe)@NC core–shell catalysts.

Ni-based catalysts have been proposed as a promising low-cost OER catalyst. While degrading easily in acidic electrolytes,^10,11^ they are quite stable in alkaline electrolytes.^11,12^ However, OER in alkaline conditions, eqs 1–4, which involves catalytic steps of OH adsorption, O–H dissociation, O–O coupling, and O_2_ evolution,^3,6^ still oxidizes metal surfaces during the catalytic reactions. After the electrochemical cycles, Ni^0^ of Ni and Ni^2+^ of Ni(OH)_2_ are oxidized into Ni^3+^ to form γ-NiOOH and then β-NiOOH (Figure 1, left),^12,13^ enhancing the OER performance.^14^ The OER performance of such NiOOH-type catalysts is further improved by alloying it with a small amount of well-dispersed Fe, i.e., Fe doping.^15−25^ Such Ni_1–*x*_Fe_*x*_OOH-type catalysts (Figure 1, left) are currently the most promising low-cost OER catalysts working in alkaline conditions.^7,8^ The potential-determining step (PDS), the active site, and the role of Fe in such Ni_1–*x*_Fe_*x*_OOH catalysts have been identified by electrochemical,^22,23^ spectroscopic,^24−31^ computational,^29−38^ and kinetic^39^ analyses, although they are still controversial^8,40−42^ and new models of active sites are still proposed and identified.^43−46^

Randomly exposed active sites of these Ni_1–*x*_Fe_*x*_OOH catalysts, however, are unstable and eventually dissolve when exposed to harsh electrolytes, keeping these catalysts still inappropriate for long-term operations.^45−47^ Recently, their stabilities and activities have been improved by encapsulating the catalytic nanoparticles with thin graphitic layers, which expose only an optimum amount of active sites to electrolytes (Figure 1, right). For example, a Ni–Fe bimetallic analogue of Fe(III)_4_[Fe(II)(CN)_6_]_3_ Prussian blue has yielded high-activity-high-stability hybrids between NiFe metal cores and N-doped carbon (NC) shells (NiFe@NC) via a simple pyrolysis, during which the nitrile ligands decompose and form the NC layers encapsulating the metal in core–shell nanostructures.^48−50^ A number of other NiFe@NC core–shell catalysts^51−73^ have also shown enhanced OER activities and stabilities.

However, the exact OER mechanism of these catalysts as well as the exact roles of core–shell encapsulation, Fe doping in the core, and N doping in the NC shell are still unclear. Even the exact location of the OER active site is still under debate^54^ between the core^48,56−59,62,66,68,69^ and the shell.^51,53,55,63,64,71^ Since the OER activity is completely lost with the NC shells alone (e.g., after washing away core metals by acids),^48,55,56,58,62,66,68^ the role of the NC shell may be simply to enhance the electrical conductivity between the active core nanoparticles^61,62^ or to protect the active metal cores from excessive oxidation, dissolution, or agglomeration by exposing only an optimum amount of active sites on the core surfaces to harsh electrolytes.^48,58,62,69^ The NC shell may also indirectly alter the electronic structure of the active site on the enclosed core^48^ or promote their binding to key intermediates.^60^ On the other hand, the NC shells, after electron penetration or metal atom (M) dispersion (as M–N–C bridges) from the metal cores, can also actively participate in the key OER steps, serving as the active site.^51,53,56,62−66,70,71^

Therefore, the key questions concern the exact structure of NiFe cores and NC shells at their interface, the OER energetics on these models, and which one, the core or the shell, constitutes the active site that requires the lowest overpotential. However, previous density functional theory (DFT) studies on the NiFe@NC core–shell catalysts or their derivatives have assumed simple models such as purely metallic cores or pure graphene shells,^51,55,67,68,72^ while various experiments indicate that rather complex active sites develop at the core–shell interface during synthesis or activation of these catalysts. Therefore, herein, we first derive plausible computational models of the active site on each component (core or shell) based on various experimental evidence and our DFT calculations. We then estimate the OER energetics of these active-site models to determine which component serves as the active site and what is the role of the other component to enhance the OER performance. We finally conclude that the major active site would be on the NiFe cores but the NC shells would provide not only a protection of the vulnerable active sites on the cores against dissolution but also an independent active site in the form of NiN_4_C.

## 2 Calculation Details

The OER free-energy diagrams are built by calculating the reaction free energy of each step producing a series of OER intermediates (OH*, O*, OOH*, and OO*, and finally, O_2_; eqs 1–4) in the alkaline condition of pH 14. To avoid unreliable calculations of isolated intermediates and products (OH, O, and OOH radicals and triplet O_2_), these free-energy changes (or costs; ΔΔ*G*) are defined with respect to the computational hydrogen electrode (pH 0), i.e., in terms of rather reliable energies of isolated H_2_O and H_2_,^74−79^ which are calculated once by DFT geometry relaxation at the Γ point in a large periodic cubic unit cell with a side length of 20 Å
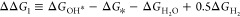
5
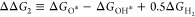
6
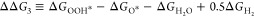
7
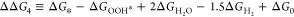
8where Δ*G*_0_ in eq 8 is taken from experiments (4.92 eV).^78,79^ The energies of bare (E*) and adsorbed (*E*_OH*_, *E*_O*_, and *E*_OOH*_) catalytic surfaces (core or shell) are first calculated on various adsorption sites and at various stages (including reconstruction and oxidation) along the OER cycles. The most stable adsorption state with the lowest energy *E* is selected at each stage. Energy costs (Δ*E*) are first estimated between consecutive states and then converted to the free-energy costs ΔΔ*G* by adding various corrections (ΔΔ*G*_corr_) concerning zero-point energy, entropy at 298 K, and pH at 14.^74−77^ The amounts of these corrections on ΔΔ*G*_1_ to ΔΔ*G*_4_ are −0.43, −1.20, −0.40, and −1.29 eV.^29,55,75,77,80^ The reaction step requiring the largest ΔΔ*G* among them is chosen as the PDS. This maximal free-energy cost ΔΔ*G*_max_ can be used to estimate the overpotential η,^76^ but, due to the limited accuracy of ΔΔ*G*_corr_, we focus on relative values rather than absolute values. The calculations are performed by spin-polarized DFT with the Perdew–Burke–Ernzerhof (PBE) exchange–correlation functional,^81^ the Grimme D3 dispersion correction,^83^ a kinetic energy cutoff of 600 eV, and the projector augmented wave (PAW) pseudopotentials implemented in VASP.^84^ An energy convergence criterion of 0.01 eV Å^–1^ is set for the geometry relaxation. The energies are calculated with the 6 × 6 × 1 (NiFe core) or 3 × 3 × 1 (NC shell) Monkhorst–Pack *k*-point mesh^85^ after relaxing the positions of all of the atoms except those in the bottom layer of the NiFe core. [A Hubbard *U* correction (DFT+*U*) calculation with the Dudarev formalism,^86^*U*–*J* terms of 5.5 (Ni) and 5.3 (Fe) eV,^87,88^ and full geometry relaxation is shown only in the Supporting Information].

## 3 Results and Discussion

### 3.1 Active Site on NiFe Core

**Figure 2:**
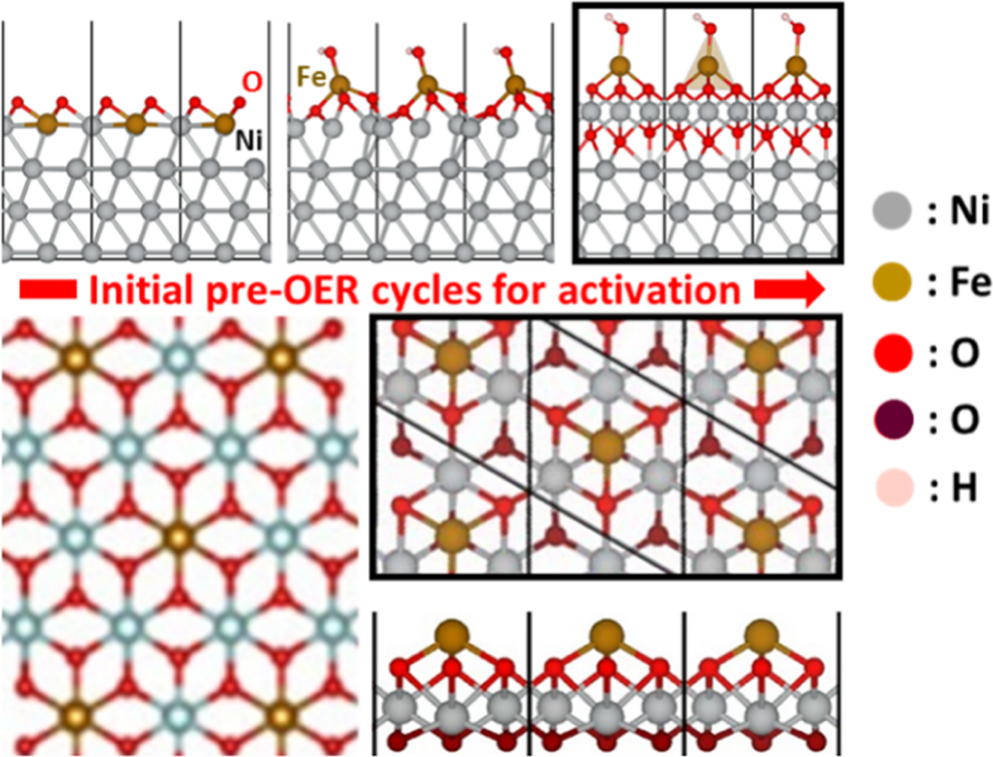
Active-site model for the NiFe core side of the NiFe@NC catalyst. The top and side views on its top oxide layer are quite comparable to those of a Ni_1–*x*_Fe_*x*_OOH structure suggested by an XAS experiment (bottom left; reproduced from ref (8). Copyright 2023 American Chemical Society).

We herein avoid the purely metallic Ni^0^/Fe^0^ model typically used for encapsulated catalysts^51,55,67,68,72^ and the Ni_1–*x*_Fe_*x*_OOH model typically used for fully exposed catalysts.^29−32,34−38,58^ Instead, we take an active-site model constituted of an Fe atom sitting on a Ni oxide layer (in the form of FeO_4_ or more specifically FeO_*x*_H_*y*_; Figure 2), which was grown on the fcc-NiFe (111) surface under a strong alkaline condition computationally (i.e., by finding the lowest-energy structure as a series of OH is sequentially introduced to the surface).^100^ Indeed, most X-ray diffraction (XRD) experiments on core–shell catalysts have identified an fcc crystal structure for NiFe metal core before OER operation, and most X-ray spectroscopy (XPS) studies have shown dominant peaks for metallic Ni^0^/Fe^0^ on the core surface (unless synthesized with KOH activation),^66^ which are gradually oxidized to Ni^2+^/Fe^3+^ during the electrochemical cycles.^48−65,67−72^

Each layer (including the topmost Fe/Ni_3_ layer) in the unit cell (*a* = *b* = 4.925 Å, *c* = 21.337 Å) has four Ni atoms (Ni_16_). The Fe concentration, 25 atom % (Ni_3_Fe_1_) at the top surface and 6.25 atom % (Ni_15_Fe_1_) in the whole slab, can be considered to cover the Fe doping in typical high-performance NiFe@NC catalysts (5–25%).^47^ The protrusion of oxophilic Fe as FeO_4_ is more stable than the alternative protrusion of Ni as NiO_4_. The first oxide layer under the protruded Fe atom contains, per unit cell, three remaining Ni atoms and six O atoms connecting four metal atoms under them. The optimized distances (Å) of Fe–O (1.78–1.93), Ni–O (1.90–1.92), and Fe–Ni (2.73–2.76) are comparable to an X-ray absorption spectroscopy (XAS) measurement (Fe–O 1.82–1.98, Ni–O 1.89–1.92, and Fe–Ni 2.81–2.97).^29^ The Fe–O–Ni arrangement in our surface layer is quite similar to those suggested by a number of experiments.^7,8^ The only difference is that our Fe atom sits on the intact Ni oxide layer instead of replacing one of the Ni atoms in that layer, but the limited solubility of Fe in Ni (oxy)hydroxide suggested by previous experiments^29^ supports our Fe-protruded surface model.

Such FeO_4_-type active sites sitting on Ni oxide layers are supported by a number of experiments suggesting dynamically stable^45^ Fe active sites incorporated onto a stable host of Ni(OH)_2_ or NiOOH.^22,23,43−47,89^ They are also supported by experimental observations of selective Fe dissolution (probably in the form of Fe(OH)_4_^–^, FeO_2_^–^, FeO_4_^2–^, or FeO_*x*_H_*y*_ in general) from NiFe-based OER catalysts and concomitant activity reduction, especially in Fe-free KOH electrolytes.^44−47,89^ They are also consistent with the activity enhancement observed immediately after spiking Fe impurities into electrolytes [probably in the form of Fe(OH)_4_^–^ at pH 14].^16,22,23,43−47,89^

### 3.2 Active Site on NC Shell

For the NC shell, considering the metal binding ability of N, it is plausible^56,66,67^ that the synthesis of NiFe@NC by a pyrolysis of NiFe/N/C-containing precursors would result in a group of (most likely four) N atoms coordinating a single Ni or Fe atom at least in the NC layer adjacent to the metal core. Such a porphyrin-like metal-bound 4N-graphene (MN_4_C; M = Ni or Fe) single-atom catalyst (SAC) has shown excellent OER activities.^90,91^ In fact, these MN_4_C SAC catalysts and the M@NC core–shell catalysts have similar compositions (M, N, and C) and both are synthesized by similar heat treatments at similar temperatures (≤900 °C). A majority of metal atoms would aggregate into the metallic cores, but a small amount of metal atoms may still move around in the adjacent NC layers and get trapped in N_4_C vacancies to form MN_4_C.^92^ Indeed, the N 1s XPS spectra of both systems show peaks at the same positions,^50,90^ i.e., peaks for N–Ni along with the peaks for pyridinic N and graphitic N,^93^ revealing similarities between Ni@NC and NiN_4_C SAC. The M 2p XPS spectra also suggest that both systems have similar M-coordinating N and O environments.^60,67,90,91^ The M–O peaks most likely originate from metal oxidation, but the M–N peaks may indicate the presence of SAC-like MN_4_C coordination in the M@NC catalyst. The energy-dispersive X-ray spectroscopy (EDX) elemental mapping has also indicated a homogeneous distribution of C, N, O, and Ni atoms in the NC shell region.^66^ The uniformly distributed Ni is often adjacent to N, suggesting Ni–N bonding. The Ni–N coordination in NC layers has also been confirmed by extended X-ray absorption fine structure (EXAFS) analyses,^94^ and the Ni–N bond lengths of NiFe@NC are similar to those of Ni-coordinated phthalocyanine.^93^ In addition, a metal atom binding to a pyridinic N atom on the edge or in the middle of a graphene has been calculated to be favorable.^80^ On the other hand, peaks assigned to Fe–O/N/C (∼1.5 Å) are very small in contrast to the major peak (∼2.2 Å) corresponding to crystalline metallic Fe, suggesting the presence of only a negligible amount of Fe atoms in the NC shell. In fact, Ni atoms are more prone to coordinate with N-containing ligands than Fe atoms.^52^

**Figure 3:**
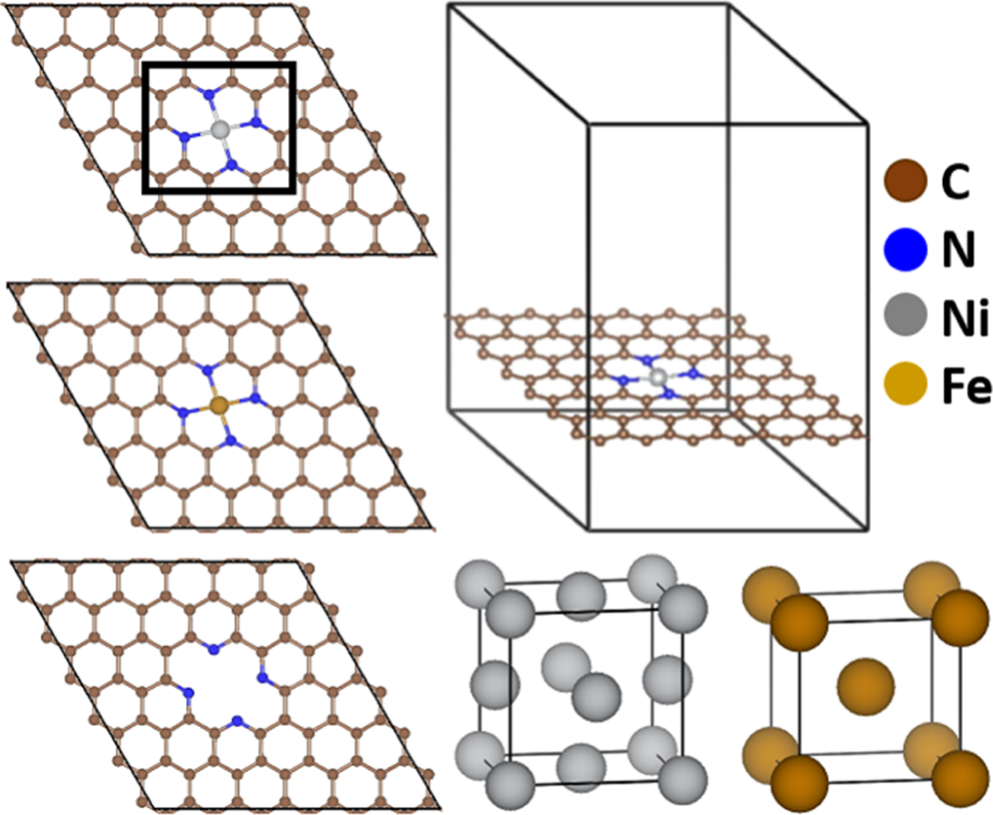
Active-site model for the NC shell of the NiFe@NC catalyst. Ni/Fe-intercalated single-atom catalyst (NiN_4_/FeN_4_; top/middle) embedded in a periodic single-layer graphene, whose plausibility is estimated by energy changes upon Ni/Fe intercalation from the bulk (fcc-Ni/bcc-Fe; bottom right) to the center of empty N_4_ site (bottom left).

Therefore, we herein estimate the plausibility of MN_4_C SAC formation in the NC shell during pyrolysis. We calculate the preference of a metal atom (M = Ni or Fe) intercalating into the vacant N_4_C site of the shell to form MN_4_C (*E*_MN_4_C_) rather than remaining in the bulk metal in the core (*E*_M_) and leaving the N_4_C center vacant (*E*_N_4_C_) (Figure 3). When the metal intercalation energy (Δ*E*_in_ = *E*_MN_4_C_ – *E*_M_ – *E*_N_4_C_) is more negative, the MN_4_C SAC formation in the shell is more plausible. *E*_M_ is the energy per atom of the bulk metal (fcc-Ni or bcc-Fe; Figure 3, bottom right) fully optimized with a 12 × 12 × 12 Monkhorst *k*-point grid. The NC shell with or without the intercalated metal atom M is represented by a minimal (M)N_4_C SAC model, where four pyridinic N atoms are embedded in a (6 × 6) hexagonal supercell of free-standing single-layer graphene composed of 72 C atoms (*a* = *b* = 14.81 Å, *c* = 25 Å; Figure 3), as proposed in previous studies,^67,80,90^ either binding a single metal (M = Ni or Fe) atom in the center (*E*_MN_4_C_) or leaving the center empty (*E*_N_4_C_). This N-doping concentration (∼6 atom %) is consistent with experiments (3–16 atom %).^48,49,51,58,61−63^

The calculated metal intercalation energy Δ*E*_in_ indicates that a NiN_4_C site (Δ*E*_in_ = −4.67 eV) is much more likely to form than an FeN_4_C site (Δ*E*_in_ = 0.30 eV). Moreover, there are much more Ni atoms than Fe dopants (5–25%) available during the synthesis of typical high-performance NiFe@NC catalysts.^47^ We thus exclusively choose the NiN_4_C SAC model to study the OER on the shell.

### 3.3 Active Site of NiFe@NC: Core or Shell?

**Figure 4:**
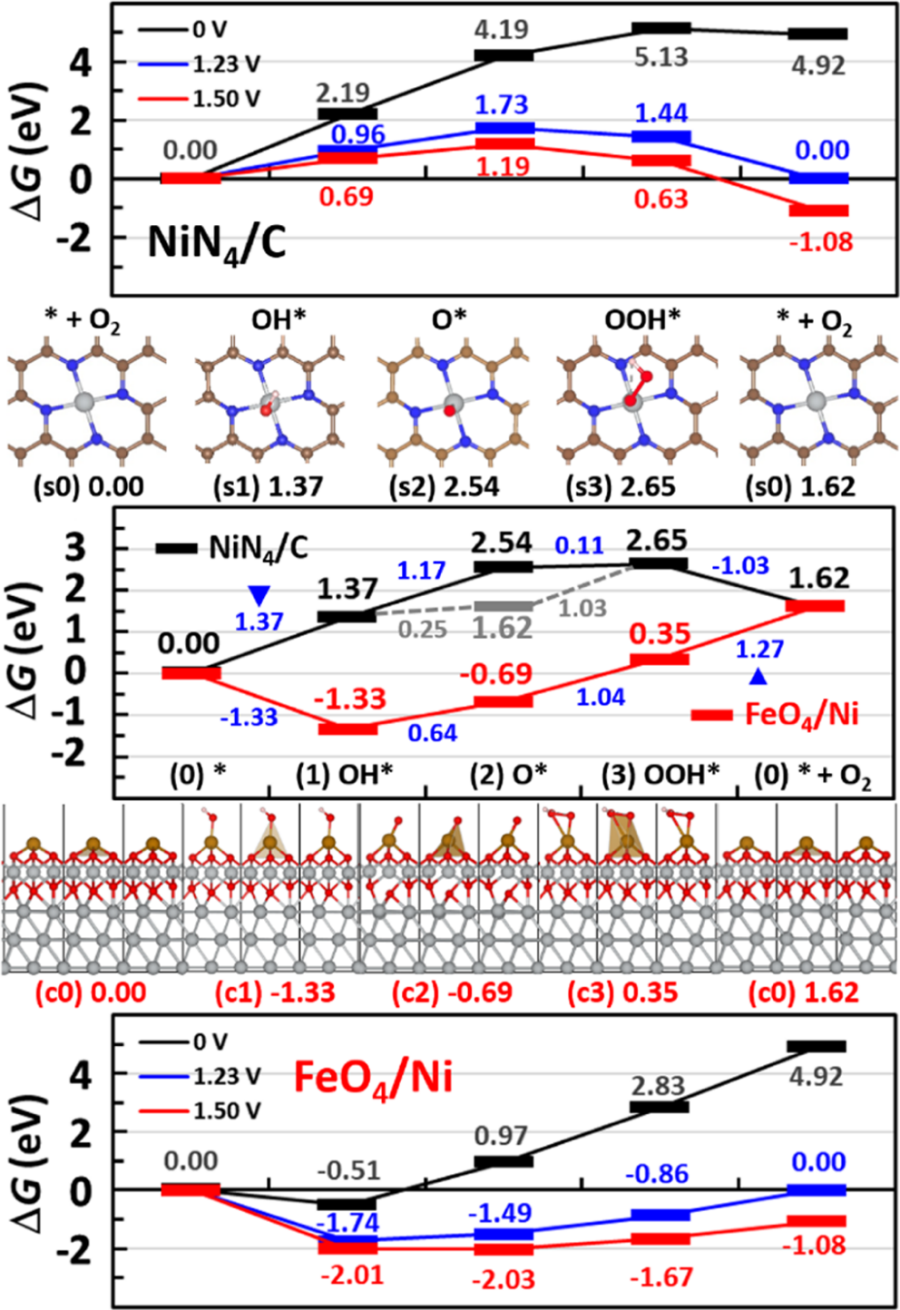
[Middle] OER free-energy diagrams built for the active-site models of NiFe core (red bar) and NC shell (black bar) [color code: Ni, gray; Fe, golden; O, red; N, blue; C, brown; and H, white] vs the standard hydrogen electrode (SHE), which is 0.826 V vs reversible hydrogen electrode (RHE) at pH 14. Free-energy changes ΔΔ*G* are shown in blue, and their maxima ΔΔ*G*_max_ are marked by blue triangles. [Top and bottom] OER free-energy diagrams presented at potentials of 0.0 (black), 1.23 (blue), and 1.50 (red) V vs RHE.

Figure 4 shows the OER free-energy diagrams built separately for the active-site models of the NiFe core (Figure 2) and the NC shell (Figure 3). On the active site of the NiFe core (Figure 4, red), the OH adsorption on the bare Fe atom site (state c0) to produce OH* (state c1) is extremely favorable/downhill (eq 1; ΔΔ*G* = −1.33 eV), probably due to the oxophilicity of Fe. Therefore, the next steps, i.e., the O–H dissociation of OH* to produce O* (state c2) and the O–O coupling to produce OOH* (state c3), are unfavorable/uphill (eqs 2 and 3; ΔΔ*G* = 0.64 and 1.04 eV). In agreement with a recent report,^79^ the most unfavorable step, PDS, turns out to be the last O_2_ evolution step (eq 4; ΔΔ*G*_max_ = 1.27 eV), i.e., the second O–H dissociation (OOH* to O_2_*) followed by the O_2_ desorption, which regenerates the bare metal atom site (*; state c0). This bare metal atom site would be immediately occupied by OH (state c1) coming from the electrolyte at pH 14 in order to be ready for the next OER cycle.

On the other hand, the OER free-energy diagram (Figure 4, black) built for the NiN_4_ SAC active-site model of the NC shell side indicates a complete shift of PDS to the first step (OH adsorption), which produces OH* either on the Ni atom of the NiN_4_ site (state s1) or on one of the C atoms next to the NiN_4_ site (not shown), with the maximal free-energy change ΔΔ*G*_max_ of 1.37 eV in both sites.

Following the lowest-energy structure of each state after that, we support a dual-site OER mechanism suggested by previous studies,^67,90^ where the second step (O–H dissociation) produces O* on one of the C atoms next to the NiN_4_ site (not shown; ΔΔ*G* = 0.25 eV) rather than on the Ni atom (state s2; ΔΔ*G* = 1.17 eV), while the third step (O–O coupling) produces OOH* on the central Ni atom (state s3; ΔΔ*G* = 1.03 eV) rather than on one of those C atoms (not shown; ΔΔ*G* = 1.46 eV).

However, considering the difficulty in hopping between different adsorption sites, we herein stick to the single-site mechanism on the central Ni atom, i.e., OH adsorption producing OH* (state s1; ΔΔ*G*_max_ = 1.37 eV), O–H dissociation producing O* (state s2; ΔΔ*G* = 1.17 eV), and O–O coupling producing OOH* (state s3; ΔΔ*G* = 0.11 eV).

The last step corresponds to the second O–H dissociation producing O_2_* on the Ni atom, which is followed by O_2_ evolution regenerating the bare active site (state s0; ΔΔ*G* = −1.03 eV). Since this bare NiN_4_ site in the NC shell is quite stable (Δ*E*_in_ ≪ 0) and hydrophobic, another OH adsorption during the next OER cycle would be again unfavorable/uphill, constituting PDS with ΔΔ*G*_max_ of 1.37 eV. This is 100 mV higher than the ΔΔ*G*_max_ value estimated for the NiFe core, implying that the major active site of NiFe@NC core–shell OER catalysts would be most likely the protruded FeO_4_-type active sites on the oxidized Ni core surface (i.e., Fe-doping effect).

The major role of the NC shell would therefore be to protect these vulnerable FeO_4_ active sites from leaching into the electrolyte and to maintain the high OER activity of the core (i.e., core–shell encapsulation effect). The NC shell is not a perfect shield that completely blocks contact between the core and the electrolyte. Vacancies and defects are enriched near the N sites and therefore more abundant in NC shells than in C shells (i.e., N-doping effect).^62,66^ Presumably, these vacancies are small enough to prevent the passage of Fe(OH)_4_^–^ for core protection but large enough to allow the passage of OH^–^ for the OER.

The NC shell would also provide independent NiN_4_-type OER sites, albeit slightly less active (i.e., another N-doping effect). Moreover, the large ΔΔ*G*_max_ value, which is ascribed to unfavorable OH adsorption to hydrophobic NiN_4_ active sites in the NC shell, may be lowered in hydrophilic environments, which can be provided by the OH* state of the FeO_4_ active site on the NiFe core. Such a mechanism corresponding to another synergetic core–shell encapsulation effect will be investigated in the near future by building OER free-energy diagrams for the core–shell composite.

## 4 Summary

Using DFT calculations, active-site models were identified for Fe-doped Ni metal cores encapsulated by N-doped graphene shells (NiFe@NC), a promising OER catalyst that works under alkaline conditions. The formation of an FeO_4_-type active site protruding from a Ni oxide layer was identified on top of the Ni core surface. Such a structure, which is attributed to the oxophilicity of Fe, has been proposed as a possible active site by recent experiments, and its facile dissolution into electrolytes explains the activity loss of such catalysts upon direct exposure to electrolytes. On the other hand, in the NC shell, the formation of a NiN_4_-type porphyrin-like single-atom-catalyst active site by favorable Ni intercalation from the core metal was identified. The OER free-energy diagrams built separately for these active-site models of the core and the shell show that the FeO_4_-type active site on the core is most likely the major active site of NiFe@NC. Labile FeO_4_-type active sites identified on top of the NiFe core surface strongly suggest the critical role played by NC shell in encapsulating them, preventing them from leaching into electrolytes, and thus maintaining the high OER activity of the core. However, the NiN_4_-type active sites identified in the NC shell are also expected to independently participate in the OER, further increasing the catalytic activity of the core–shell nanostructures.

## Supporting Information Available

Additional OER free-energy diagram calculated by DFT+*U* and the *U* parameters (PDF)
Machine-readable (XTL) DFT-optimized structures (c0–c3 and s0–s3 in Figure 4) (ZIP)
