# Supplementary material for: Active Sites of Mixed-Metal Core–Shell Oxygen Evolution Reaction Catalysts: FeO4 Sites on Ni Cores or NiN4 Sites in C Shells?
Source: ACS Omega. 2024 Jun 7;9(24):25748–55. doi: 10.1021/acsomega.3c09920 (PMC11190911; doi:10.1021/acsomega.3c09920)
Supplement: Supplementary file 1 — ao3c09920_si_001.pdf [file ao3c09920_si_001.pdf]

Supporting Information

Active Sites of Mixed-Metal Core-Shell Oxygen Evolution Reaction Catalysts. FeO_4 Sites on Ni Cores or NiN_4 Sites in C Shells?

Sung Soo Lim,[1] Arumugam Sivanantham,[1,#] Changwon Choi,[1] Sangaraju Shanmugam,[1] Yves Lansac,[1,2,3] Yun Hee Jang[1,2,*]

[1]Department of Energy Science and Engineering, DGIST, Daegu 42988, Korea

[2]GREMAN, UMR 7347, CNRS, INSA CVL, Université de Tours, 37200 Tours, France

[3]LPS, UMR 8502, Université Paris-Saclay, CNRS, 91405 Orsay, France

[#]Present Addresses: Department of Materials Science & Engineering and Department of Energy Systems Research, Ajou University, Suwon 16499, Korea

Table S1. U or U–J parameters used for DFT+U calculations on NiFeOOH derivatives.

No.	U(Ni)	U(Fe)	Reference
1a	6.6	3.5	Z. X. Shi, et al. Appl. Catal. B 2021, 298, 120558
			F. Dong, et al. Appl. Catal. B 2024, 340, 123242
			C. D. Van, et al. ACS Sustainable Chem. Eng. 2023, 11, 4, 1333-1343
1b	6.6	3.3	L. Wang, et al. ACS Appl. Energy Mater. 2022, 5, 2, 2221
2a	6.5	3.5	M. Kumar, et al. ChemPhysChem 2022, 23, e202200085
3a	6.45	5.3	C. Peng, et al. ChemSusChem 2020, 13, 811.
3a	6.2	5.3	Z. Zheng, et al. Carbon Energy 2022, 4, 901-913
			H. Liao, et al. Appl. Catal. B 2022, 317, 121713
			Z. Wei, et al., Appl. Catal. B 2023, 322, 122101
			F. Si, et al., Int. J. Hydrogen Energy 2024, 49, 143
4a	6.0	4.5	S. Hong, et al. Adv. Funct. Mater. 2023, 33, 2209543
4b	6.0	4.0	C. Zhang, et al. J. Colloid Interface Sci. 2022, 607, 967
5a	5.5	5.3	M. Liu, et al. Adv. Energy Mater. 2021, 11, 2101281
			W. Liu, et al. Chem. Eng. J. 2023, 454, 140030
			H. Chen, et al. Small 2024, 2309689
5b	5.5	5.0	H. Wang, et al. J. Catal. 2024, 430, 115354
			X. Liu, et al. Appl. Catal. B 2023, 331, 122715
5c	5.5	4.0	Y. Ou, et al. Nat. Commun. 2023, 14, 7688
5d	5.5	3.3	Y.-F. Li, et al. ACS Catal. 2014, 4, 1148
			V. Fidelsky, et al. Phys. Chem. Chem. Phys. 2017, 19, 7491
6a	4.0	4.3	Z. He, et al. Nat. Commun. 2022, 13, 2191
7a	3.8	4.3	H. Xu, et al. ACS Appl. Mater. Interfaces 2023, 15, 50, 58784
8a	3.0	3.0	J. Chen, et al. Sci. Bull. 2021, 66, 11, 1063

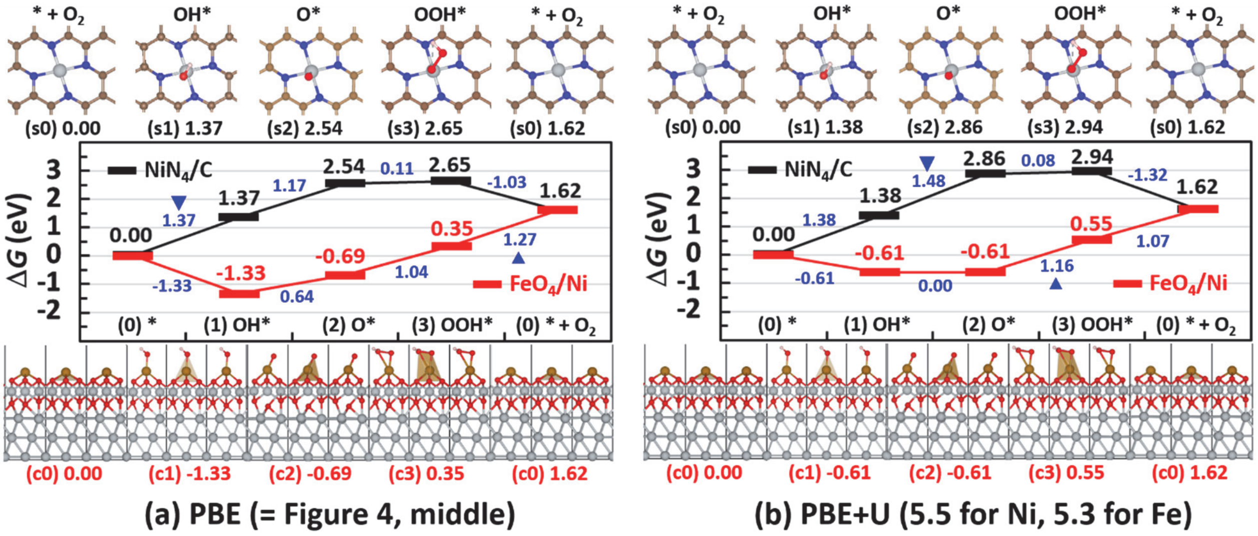

Figure S1. OER free energy diagrams calculated for the FeO_4-type active-site model of the NiFe core (red) and the NiN_4-type active-site model of the NC shell (black) without (left) and with (right) applying the DFT+U formalism of Dudarev (5.5 and 5.3 as the U–J term for Ni and Fe, respectively; in eV). Free energy changes $\Delta\Delta G$ are shown in blue and their maxima $\Delta\Delta G_{max}$ are marked by blue triangles [Color code: Ni grey, Fe golden, O red, N blue, C brown, and H white].

The Hubbard U correction or the DFT+U (or herein PBE+U) formalism is beneficial for calculations on magnetic metals and metal oxides. However, since a wide range of effective U or U–J terms have been used for DFT+U calculations on NiFeOOH-type compounds (Table S1 above), we are reluctant to introduce additional parameters to our calculations. Moreover, when the DFT+U formalism of Dudarev is applied with the most commonly-used set of effective U–J terms, 5.5 (Ni) and 5.3 (Fe), to optimize the geometries of all the states (**c0-c3** in the core and **s0-s3** in the shell, Figure S1, right), the conclusion of the PBE calculations performed without such corrections (Figure S1, left) turn out to be still valid, i.e., the maximal free energy cost $\Delta\Delta G_{max}$ is lower on the core than on the shell with or without the Hubbard U correction (core 1.27 < shell 1.37 without DFT+U in the original manuscript vs. core 1.16 < shell 1.48 with DFT+U; in eV).
