# Supplementary material for: Active Sites of Mixed-Metal Core–Shell Oxygen Evolution Reaction Catalysts: FeO4 Sites on Ni Cores or NiN4 Sites in C Shells?
Source: ACS Omega. 2024 Jun 7;9(24):25748–55. doi: 10.1021/acsomega.3c09920 (PMC11190911; doi:10.1021/acsomega.3c09920)
Supplement: Supplementary file 2 — ao3c09920_si_002.zip [file ao3c09920_si_002.zip › 2024515_SI1_cover_revision3_ao_2023_09920x_R2.docx]

**Supporting Information**

Active Sites of Mixed-Metal Core-Shell Oxygen Evolution Reaction Catalysts. FeO_4_ Sites on Ni Cores or NiN_4_ Sites in C Shells?

Sung Soo Lim,^1^ Arumugam Sivanantham,^1,#^ Changwon Choi,^1^ Sangaraju Shanmugam,^1^

Yves Lansac,^1,2,3^ Yun Hee Jang^1,2,*^

^1^Department of Energy Science and Engineering, DGIST, Daegu 42988, Korea

^2^GREMAN, UMR 7347, CNRS, INSA CVL, Université de Tours, 37200 Tours, France

^3^LPS, UMR 8502, Université Paris-Saclay, CNRS, 91405 Orsay, France

^#^Present Addresses: Department of Materials Science & Engineering and

Department of Energy Systems Research, Ajou University, Suwon 16499, Korea
